# A Systematic Review of the Amount of Water per Person per Day Needed to Prevent Morbidity and Mortality in (Post-)Disaster Settings

**DOI:** 10.1371/journal.pone.0126395

**Published:** 2015-05-11

**Authors:** Emmy De Buck, Vere Borra, Elfi De Weerdt, Axel Vande Veegaete, Philippe Vandekerckhove

**Affiliations:** 1 Belgian Red Cross-Flanders, Mechelen, Belgium; 2 Department of Public Health and Primary Care, Faculty of Medicine, Catholic University of Leuven, Leuven, Belgium; 3 Faculty of Medicine, University of Ghent, Ghent, Belgium; California Northstate University College of Medicine, UNITED STATES

## Abstract

**Background:**

In order to improve the effectiveness and efficiency of humanitarian efforts, minimum standards for humanitarian assistance and key indicators, showing whether a standard has been attained, have been developed. However, many of these standards and indicators are based on a consensus on best practices and experiences in humanitarian response, because relevant evidence on the impact of humanitarian interventions is often lacking.

**Objectives:**

One important example of a standard in humanitarian aid in a disaster setting is “water quantity.” The accompanying indicator states how many litres of water are needed per person per day in a disaster setting. It was our objective to determine the evidence base behind this indicator, in order to improve health outcomes such as morbidity (e.g., diarrhoea) and mortality.

**Methods:**

A systematic review was performed searching The Cochrane Library, Medline and Embase. We included studies performed during disasters and in refugee camps that reported a specific water amount and health-related outcomes related to water shortages, including diarrhoea, cholera, and mortality. We used GRADE to determine the quality of evidence.

**Results:**

Out of 3,630 articles, 111 references relevant to our question were selected. Based on our selection criteria, we finally retained 6 observational studies, including 1 study that was performed during the disaster and 5 studies in a post-disaster phase. From two studies there is conclusive evidence on the relationship between the amount of water received and diarrhoea or mortality rates in refugee camps. However, overall, these studies do not contain enough data with relevance to a specific amount of water, and the level of evidence is very low.

**Conclusions:**

More primary research on water amounts in a disaster setting is necessary, so that the humanitarian sector can further professionalise its water-related standards, indicators and interventions.

## Introduction

### Evidence-based approach to disaster management

When disaster strikes, the ultimate goal of any disaster management programme is to obtain the best possible outcome for the greatest number of people [1]. A rapid and coordinated response remains one of the greatest challenges [1–5]. The larger the incident, the greater the number of agencies and jurisdictions involved, all with their own routines and procedures, as they are often based on expert- or tradition-based decisions [3]. It is essential that policies and practices are based on the best available evidence in order to standardise and maximise the utilisation of available resources. Evidence-Based Practice balances the best available evidence on the effectiveness of interventions with practice experience from experts in the field, and with preferences of the target group, in this case the affected population [6]. The use of this methodology in the health sector has resulted in improved practice guidelines and more effective decision making. However, a similar approach is difficult to employ in the disaster sector, since evidence is often lacking [6–10].

In order to improve the effectiveness and efficiency of humanitarian efforts, different minimum standards for humanitarian assistance and indicators, showing whether a standard has been attained, have been developed. Today most governments and organisations rely on the Sphere Handbook or the handbook for emergencies of the United Nations High Commissioner for Refugees (UNHCR) as a reference for these standards and indicators [11,12]. However, they are rarely based on evidence [13,14], but rather upon a consensus on best practices in humanitarian assistance [11].

This points to the importance of developing systematic reviews in order to obtain an overview of the available evidence and to assess the quality of the evidence supporting standards and indicators in humanitarian aid. It also highlights the areas of humanitarian assistance in which more evidence is needed. Efforts are already being made in this area, e.g. by Evidence Aid, collecting systematic reviews relevant for the disaster setting [15,16], the Enhancing Learning and Research for Humanitarian Assistance (ELHRA) initiative, resulting in “The Humanitarian Health Evidence Review” [17], and the Belgian Red Cross, using Evidence-Based Practice to support its activities [18].

### Example on the provision of water during disaster

One example is the indicator for the amount of water necessary in disasters (litres per person per day, l/p/d), which is crucial, since the destruction of safe water supplies and sanitation facilities is considered one of the major causes of disease outbreak in the aftermath of a disaster [19–22]. For this indicator, different organisations or references recommend different amounts of water, ranging from 10 to 20 l/p/d [11,12,23–28]. Table 1 shows an overview of different indicators for the amount of water per person per day needed, used by the following organisations: Sphere, UNHCR, the Federal Emergency Management Agency (FEMA), the United States Army Corps of Engineers (USACE), the United States (US) Environmental Protection Agency (EPA) and the Office of Foreign Disaster Assistance (OFDA) [11,12,23–26]. In addition we found two other references reporting water needs per person per day [27,28].

**Table 1:** Comparison of the different minimum standards for water and sanitation needs.

	Sphere Project [11]	UNHCR [12]	EPA [23]	FEMA [24]	OFDA [25]	USACE [26]	White et al. [27]	Reed et al. [28]
**1. Drinking water needs per person per day (l/p/d)**	3–5	7	1.89*	3,79*	3–4	3	1.8–3§	3–5
**2. Domestic water needs per person per day (l/p/d)**	15	20	20.8*	NA	15–20	NA	10–20	15–20
**3. Communal latrine coverage**	20 people per latrine	20 people per latrine	NA	NA	20 people per latrine	NA	NA	NA
**4. Distance from the farthest dwelling to water point**	<500 m	<200 m	NA	NA	<100 m	NA	NA	NA
**5. Number of persons at each water point**	250 per tap500 per hand pump400 per well	80–100 per tap200–300 per hand pump/well	NA	NA	200–250 per tap	NA	NA	NA

* The original data were in gallons and were converted to litres using the following ratio: 1 gallon = 3.785 litres. NA: no information available. l/p/d: litres per person per day.

^§^ This is the amount African people went to collect, when they had to make use of a communal water source.

The most critical component in water requirements is the amount needed for drinking and eating. In the above-mentioned resources, we found amounts of drinking water varying between 1.89 to 7.0 l/p/d (see Table 1). Most of these indicators are based on assumptions for disaster settings or emergencies [11,12,23–26,28]. White et al. described the relationship between the minimum daily requirement of water, the climate and the effect of activity [27]. He concluded that the minimum drinking water requirement for survival in a tropical area falls in the range of 1.8 to 3.0 l/p/d, and that daily use of water ranges from 10 to 20 l/p/d when supplied by a standpipe in the vicinity [27].

Domestic water needs also cover basic hygiene practices and basic cooking practices. Separate data about these needs are not discussed in detail in the literature, in contrast to the average needs for global domestic use. Six different agencies define minimum levels, ranging from 10 to 20.8 l/p/d (see Table 1). None of these indicators seemed to be based on solid evidence.

Other factors also play a role in morbidity and mortality rates due to diarrhoea and other water-related diseases, for example the distance to a water source, the availability of latrines and the number of people per water source. Only 3 agencies offer a standard for these aspects, in the aftermath of a disaster, but again there is no consensus on the exact numbers, and it is not clear how the data were obtained [11,12,25].

### Objectives

In this paper we describe the results of our systematic review of the evidence supporting the indicator for water amounts, in order to improve health outcomes such as morbidity (e.g. diarrhoea) and mortality. Our PICO question was formulated as follows: “For survivors of disasters (Population) how many litres of water per person per day (Intervention) are necessary to minimise adverse health effects (Outcome) compared to another amount (Comparison)?”

## Methods

We followed the PRISMA statement for the reporting of this systematic review [29]. No protocol for this systematic review existed or was published beforehand. The PRISMA checklist can be found in S1 Appendix.

### Selection criteria

We used the following inclusion and exclusion criteria for selection of articles:

Population: Inclusion: Studies conducted in disasters and refugee camps were included. Refugee camps were included because we also want to obtain information for an indicator in the post-emergency phase.

Intervention/Risk factor: Inclusion: For a study to be included, provision of a certain amount of water to a population in disaster settings or in refugee camps should be described. The exact amount of water or the use of water containers had to be mentioned in the article, otherwise the article was excluded.

Comparison: Inclusion: provision of another amount of water, or no water provision.

Outcome: Inclusion: The outcome measures were health-related outcomes related to water shortages, such as diarrhoea, communicable diseases, infectious diseases, dehydration and malaria.

Study design: Inclusion: Intervention studies: randomised controlled trials, controlled clinical trials, before- and after studies; Observational studies: cohort studies, case-control studies, cross-sectional studies; Exclusion: non-controlled studies, case reports, case series, letters, comments, opinion pieces, narrative reviews.

Language: We included studies in English, French, Dutch and German.

### Search strategy and study selection

The following databases were searched from their date of inception to 22 September 2014: MEDLINE (using the PubMed interface), Embase (using the Embase.com interface) and The Cochrane Central Register of Controlled Trials. Full details of the search strategies are given in S2 Appendix. Study selection was performed in parallel by two independent reviewers (EDB and EDW, or EDB and VB). Titles and abstracts of the studies identified by the search were scanned. When a relevant article was found, full text articles were retrieved. Studies that did not meet the inclusion and exclusion criteria were excluded. The citation and reference lists of included studies were searched, and the first 20 related items in PubMed were scanned for other potentially relevant studies. Any discrepancies among the reviewers were resolved by consensus.

### Data collection

Data concerning study design, study population, outcome measures (expressed as mean difference, risk ratio or odds ratio), and study quality were extracted independently by two reviewers (EDB, EDW and VB). P-values were taken directly from the individual studies, unless it was indicated that the mean difference, confidence interval and p-value was calculated by the reviewer(s) using Review Manager software. In the event of missing data, the authors were contacted for more detailed information. No statistical methods were used to pool the data because of heterogeneity of the studies. Review Manager was used to calculate effect measures, if not reported in the study.

### Quality of evidence

The GRADE approach was used to assess the overall quality of evidence included in this review. Limitations in study design were analysed at the study level using the items listed by GRADE [30].

## Results

### Study selection

**Fig 1:**
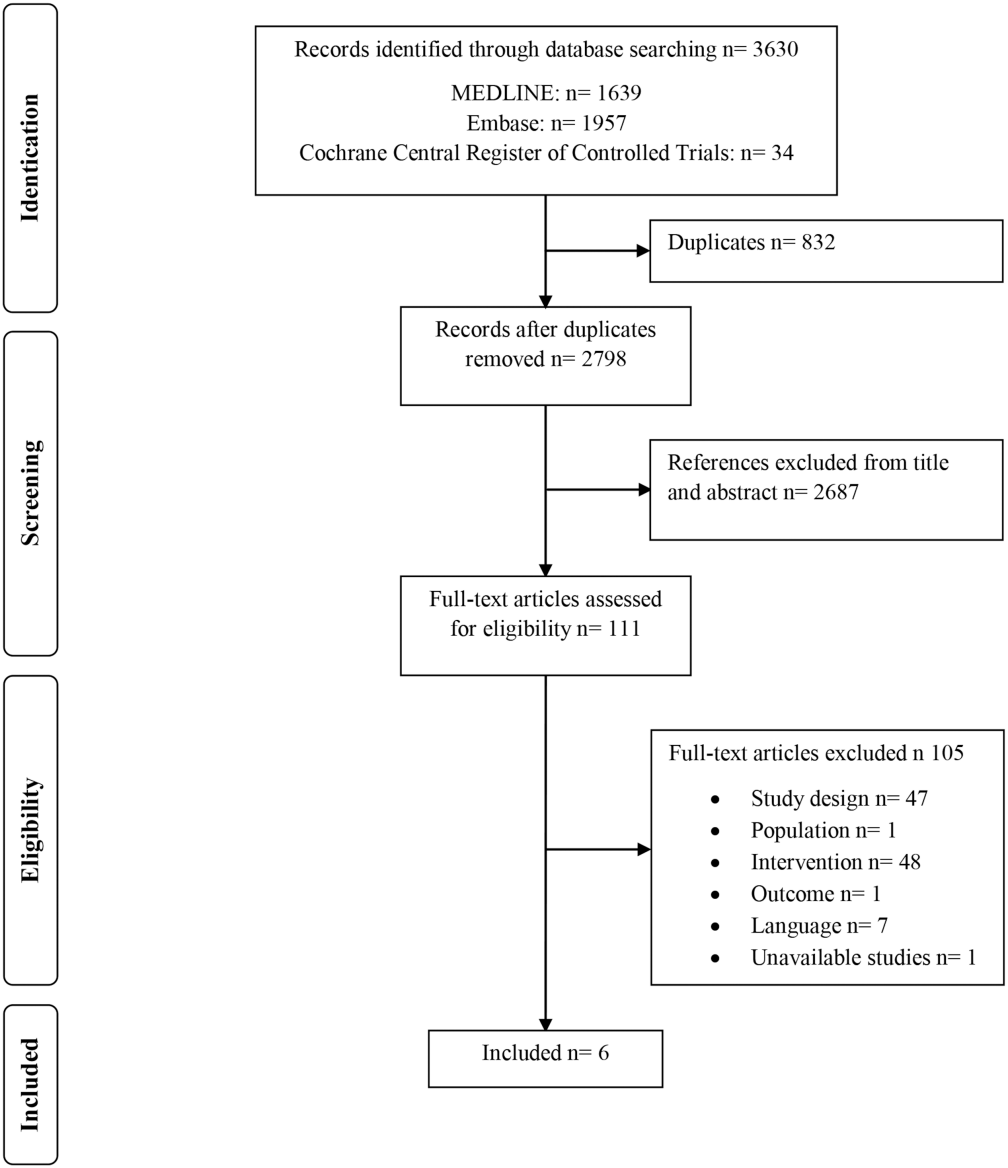
PRISMA flowchart of identification and selection of studies.

A flowchart showing study selection is given in Fig 1. 3630 citations, including 832 duplicates were found. Evaluation of titles and abstracts resulted in 111 references; 2687 studies did not answer our PICO question and were therefore ineligible. After full text evaluation, 105 studies were excluded because they did not meet the inclusion criteria.

47 studies did not meet the inclusion criteria for study design. Some of these articles were opinion pieces, narrative reviews or overviews of interventions that were applied during disasters. Approximately half of the studies were excluded because they did not mention the exact amount of water or the use of a water container. In S3 Appendix an overview is given of all studies that were excluded with their reason for exclusion. Only 6 studies met all the inclusion criteria and were available for analysis.

### Study characteristics

Five out of 6 studies were performed in the post-disaster phase (refugee camps) [31–35] and only 1 study was performed during the disaster phase [36]. All studies were observational studies, including 2 case-control studies [32,35] and 4 cross-sectional studies [31,33,34,36].

Cronin et al. describe a cross-sectional study performed in 2 refugee camps, one in Ghana and one in Kenya. 840 households were interviewed in Ghana and 285 households in Kenya. The same questionnaire was used in both refugee camps. Two important parameters they used were (i) cases of diarrhoea and (ii) the amount of water consumed per person per day [31].

One case-control study is performed by Hatch et al. in a refugee resettlement in Malawi. The characteristics of 48 households with any member(s) hospitalised for suspected cholera were compared with 441 control households. This study compares the presence of any watertight container, which was used by families to transport water (‘water container’), with the absence of water containers. The water container had a minimum capacity of 10 litres and the lack of a water container is seen as a proxy for inadequate volumes of drinking water [32].

The second case-control study is performed by Mahamud et al. in a refugee camp in Kenya. This study compared potential risk factors, such as food, water, sanitation and hygiene practices among 93 hospitalised cases of watery diarrhoea (≥ three watery stools in 24 hours) and 93 matched controls. The quantity of water consumed per person per day was calculated for both cases and controls [35].

Roberts et al. performed a randomised intervention trial in a Mozambican refugee camp in Malawi, with 1160 refugees. The aim of this randomised intervention trial was to measure the health impact of improved buckets, 20-litre water containers with a cover and a spout to prevent household contamination of water, compared to standard buckets. We did not include these data in our systematic review, but we did include the data about the relationship between diarrhoea and “possessing a water container in the household”; however these were secondary observational results (based on a questionnaire) and the raw data were not available. After contacting the author, more data were provided, but not sufficient to be able to calculate the effect measure [33].

The cross-sectional study of Spiegel and colleagues is performed in 51 post-emergency camps in 7 different countries (Azerbaijan, Ethiopia, Myanmar, Nepal, Tanzania, Thailand and Uganda). It is a retrospective study which looked at the relationship between mortality data of the last 3 months and water consumption (calculated by dividing the daily quantity of water supplied to the camp by the total number of people) [34].

The only study performed in a disaster setting is that by Thacker et al. in Haiti. Because of a drought some parts of Haiti were without electricity and as a consequence without water in areas dependent on water supplied by electric pumps. After the drought, a questionnaire asking for different health outcomes was filled in by people from an area with normal water supply (more than 1 can per person per day) and from an area without water supply (less than 1 can per person per day). The volume of 1 can was estimated to be 18.9 l [36].

An overview of the study characteristics is given in Table 2. In summary, there is a lot of heterogeneity between the different studies, at the level of the population (different populations, disaster settings, climate), the level of the intervention (different aspects of water supply: source, quality, distribution, utilisation, amount and the way the amount was measured), and at the level of outcomes (diarrhoea, cholera, mortality).

**Table 2:** Characteristics of the studies included.

Author, year Country	Study design	Population	Comparison/Risk factor	Remarks
Cronin, 2008, Switzerland [31]	Cross-sectional study	840 refugee households were interviewed in Ghana (123 households reported cases of diarrhoea) and 285 refugee households were interviewed in Kenya (47 households reported cases of diarrhoea)	Different average amounts of water usage a day	839 and 283 households were used to calculate the data, respectively
Hatch, 1994, USA [32]	Case-control study	A total of 489 refugee households were interviewed in Malawi. 48 suspected cholera households were compared with 441 control households	No water container versus any water container	The lack of water containers is seen as a proxy for inadequate volumes of drinking water (families without any water container(s) would not be likely to have access to the recommended volume of water)
Mahamud, 2012, Kenya [35]	Case-control study	93 hospitalised diarrhoea cases and 93 matched controls in Kakuma refugee camp in Kenya were interviewed	Quantity of water consumed per person per day	This study tries to identify possible risk factors for cholera.
Roberts, 2001, USA [33]	Observational study	310 out of the 1160 Mozambican refugees in Malawi received an improved bucket.	Risk factor “buckets in household” (versus no buckets in household); different amounts of water used per day were compared (additional data from author)	The study is originally a randomised intervention trial, studying the effect of an improved bucket versus a standard bucket. However, these data were not extracted for the purpose of this systematic review. We only included data from the observational part of the study (based on a questionnaire). After contacting the author, additional data were provided.
Spiegel, 2002, USA [34]	Cross-sectional study	678 296 people were included from 51 post-emergency refugee camps. Azerbaijan: 7 camps (19200 refugees); Ethiopia: 11 camps (238220 refugees); Myanmar: 3 camps (7700 refugees); Nepal: 7 camps (98100 refugees); Tanzania: 7 camps (171021 refugees); Thailand: 5 camps (30176 refugees); Uganda: 11 camps (113879 refugees)	Refugees who received <15 l/p/d (12 camps) versus refugees who received 15-20l/p/d (12 camps) versus refugees who received >20 l/p/d (27 camps)	
Thacker, 1980, USA [36]	Cross-sectional study	3929 drought-affected people were included, including 1997 from an area with normal water supply versus 1932 from an area with restricted water supply	<18.9 l/p/d versus >18.9 l/p/d	

### Synthesis of findings

An overview of the synthesis of findings of all the studies included can be found in Table 3.

**Table 3:** Synthesis of findings.

Outcome	Comparison	Effect Size	# participants	Author, year
Average amount of water (l) per person per day	Households reporting cases of diarrhoea versus households reporting no cases of diarrhoea	Statistically significant: Ghana: 30.9 ± 3.4 versus 41.8 ±2.2 versus; MD: -10.90 95% CI [-11.52;-10.28] (p<0.05); Kenya: 15.9 ± 1.3 versus 21.5 ± 1.7; MD: -5.60 95% CI [-6.03;-5.17] (p<0.05) *	839 households in Ghana and 283 households in Kenya	Cronin, 2008 [31]
Average amount of water (l) per person per day	Cases with diarrhoea versus cases without diarrhoea	9.8 versus 12.2; MD: -2.4; Not enough data available to calculate CI ^†^; (p<0.05)	186 ^§^	Mahamud, 2012 [35]
Risk of cholera	One or more water container versus no water container	Statistically significant: aOR: 0.02, 95% CI [0.003;0.12] (p<0.05)	489 households	Hatch, 1994 [32]
Risk of diarrhoea in children younger than 5	One or more water container versus no water container	RR: 0.86 (p>0.05); Not enough data available to calculate CI ^†^	1160	Roberts, 2001 [33]
Risk of diarrhoea in children younger than 5	Different amounts (l/p/d): <10, 10–15, 15–20, 20–30, >30	Incidence: <10: 260/1000; 10–15: 670/1000; 15–20: 590/1000; 20–30: 410/1000; >30: 250/1000; Not enough data available to calculate RR ^†^; Statistically significant: 10–15 versus >30; RR: 2.5; Not enough data available to calculate CI ^†^ (p<0.05, reported in Roberts 2001)	1160	Additional data from author
Risk of diarrhoea (all ages)	One or more water container versus no water container	Statistically significant: RR: 0.85 (p<0.05); Not enough data available to calculate CI ^†^	1160	Roberts, 2001 [33]
Risk of diarrhoea (all ages)	Different amounts (l/p/d): <10, 10–15, 15–20, 20–30, >30	Incidence: <10: 140/1000; 10–15: 270/1000; 15–20: 220/1000; 20–30: 210/1000; >30: 110/1000; Not enough data available to calculate RR ^†^	1160	Additional data from author
<5 Mortality rates	1: >20 l/p/d vs 2: 15–20 l/p/d vs 3: <15 l/p/d	Statistically significant: 3 versus 1: RR: 5.31, 95% CI [1.46;19.35] (p<0.05); 2 versus 1: RR: 5.24, 95% CI [1.49;18.49] (p<0.05)	678296	Spiegel, 2002 [34]
Crude mortality rates	1: >20 l/p/d vs 2: 15–20 l/p/d vs 3: <15 l/p/d	Not statistically significant: 3 versus 1: RR: 1.30, 95% CI [0.60;2.84] ^¥^ (p>0.05); 2 versus 1: RR: 1.12, 95% CI [0.57;2.21] ^¥^ (p = 0.76)	678296	Spiegel, 2002 [34]
Number of children with one or more disease, in families with low socio-economic status	<18.9 l/p/d versus >18.9 l/p/d	39.6% versus 19.5% (p>0.05); Not enough data available to calculate CI ^†^	3929	Thacker, 1980 [36]
Illness rates <6 years in families >4 persons	<18.9 l/p/d versus >18.9 l/p/d	Statistically significant: 51.6% versus 33.0% (p<0.05); Not enough data available to calculate CI ^†^	3929	Thacker, 1980 [36]
Diarrhoea rates	<18.9 l/p/d versus >18.9 l/p/d	28.7% versus 25.5% (p>0.05); Not enough data available to calculate CI ^†^	3929	Thacker, 1980 [36]
Scabies rates	<18.9 l/p/d versus >18.9 l/p/d	8.4% versus 5.0% (p>0.05); Not enough data available to calculate CI ^†^	3929	Thacker, 1980 [36]
Conjunctivitis	<18.9 l/p/d versus >18.9 l/p/d	8.0% versus 7.2% (p>0.05); Not enough data available to calculate CI ^†^	3929	Thacker, 1980 [36]
Febrile illness	<18.9 l/p/d versus >18.9 l/p/d	32.5% versus 27.4% (p>0.05); Not enough data available to calculate CI ^†^	3929	Thacker, 1980 [36]
Malnutrition	<18.9 l/p/d versus >18.9 l/p/d	8.5% versus 4.7% (p>0.05); Not enough data available to calculate CI ^†^	3929	Thacker, 1980 [36]
Families with an unemployed head of household	<18.9 l/p/d versus >18.9 l/p/d	No raw data available (p>0.05) ^†^	3929	Thacker, 1980 [36]

Raw data are presented as mean±SD (standard deviation), unless otherwise indicated. MD: mean difference; RR: risk ratio; aOR: adjusted odds ratio; CI: confidence interval.

*Mean difference (MD), confidence interval (CI) and p-value were calculated by the reviewer(s) using Review Manager software

^¥^ Imprecision (large variability of results)

^†^ Imprecision (lack of data)

^§^ Imprecision (limited sample size)

The study of Cronin et al. [31] compared the average amount of water per person per day in households with cases of diarrhoea, with the average amount in households without cases of diarrhoea. For the refugee camp in Ghana, the average amount used by refugees with reported cases of diarrhoea was 30.9 ± 3.4 l/p/d. When compared with the amount of water used by refugees reporting no cases of diarrhoea 41.8 ± 2.2 l/p/d, the mean difference was -10.90 l/p/d (95% CI [-11.52;-10.28]), which was statistically significant (p<0.00001). In Kenya, the average amount of water was lower than in Ghana. There was an average amount of 15.9 ± 1.3 l/p/d reported when cases of diarrhoea were observed and an average amount of 21.5 ± 1.7 l/p/d reported with no cases of diarrhoea. The statistically significant mean difference in this case was -5.60 l/p/d (95% CI [-6.03;-5.17], p<0.00001). In conclusion, the study showed that there is a statistically significant correlation between higher amounts of water and a decreased risk of diarrhoea. However, a causal relationship cannot be concluded since the study design is cross-sectional.

The case-control study of Hatch et al. [32] showed that the use of 1 or more water containers can be considered as a preventive measure in cholera outbreaks (aOR: 0.02, 95% CI [0.003;0.12]). This result was statistically significant (p<0.001).

In the case-control study by Mahamud et al. it was calculated that cases consumed less water per person per day than controls: 9.8 l/p/d versus 12.2 l/p/d respectively (p = 0.039), with a mean difference of -2.4 l/p/d. Confidence intervals could not be calculated due to incomplete data [35].

Roberts et al. [33] studied the risk of diarrhoea (in children younger than 5 and in the whole population) and the relationship with the use of a water container (versus no water container) and different amounts of water. In this study it was seen that the incidence of diarrhoea in children younger than 5 years decreases when the amount of water per person increases. The same effect was observed for the incidence of diarrhoea in the whole population (see Table 3). The use of a water container in the household decreased the risk of diarrhoea in the whole population by about 15% (p = 0.021), in comparison with not using a water container. The result was not significant for children younger than 5 (p = 0.222). In the additional data provided by the author and in an opinion piece for the International Committee of the Red Cross, it was reported that Mozambican refugees who received less than 15 l/p/d have a 2.5 times higher chance of diarrhoea than people who received more than 30 l/p/d, which was reported to be statistically significant for the whole population [37]. However, because of a lack of raw data we were not able to confirm or recalculate these results.

Spiegel et al. [34] investigated the effect of different amounts of water on mortality rates, comparing 3 groups of refugees who used: (i) <15 l/p/d; (ii) 15–20 l/p/d; and (iii) >20 l/p/d. After comparing <15 l/p/d with >20 l/p/d, a relative risk of 5.32 (95% CI [1.46;19.35], p = 0.03) was observed for the mortality rates in children younger than 5. Approximately the same effect was observed when 15–20 l/p/d was compared with >20 l/p/d; again this was a significant result (RR: 5.24; 95% CI [1.49;18.49], p = 0.03). For the mortality rates of the whole population, no significant results were observed: <15 l/p/d compared with >20 l/p/d resulted in an RR of 1.30 (95% CI [0.60;2.84], p = 0.76) and 15–20 l/p/d compared with >20 l/p/d resulted in an RR of 1.12 (95% CI [0.57;2.21], p = 0.76).

In the study by Thacker et al., describing a drought in Haiti, the occurrence of several pathologies associated with a lack of water was compared in people who used a water amount of more than 18.9 l/p/d versus less than 18.9 l/p/d [36]. One significant result was found in this study: the disease rates in children younger than 6 in families with more than 4 people were higher (51.6%) if they consumed less than 18.9 l than if they consumed more than 18.9 l (33.0%) (p<0.02). However, due to incomplete data the confidence interval could not be calculated. Diarrhoea rates, scabies rates, conjunctivitis, febrile illness, and malnutrition were also compared; however, these results did not differ significantly.

### Quality of the evidence

All studies included were observational studies, which results in an initial ‘low level of evidence’ according to the GRADE approach.

In all the studies risk of bias was found because of limitations in design. For 3 studies it was unclear whether the appropriate eligibility criteria were used [32–34]. Hatch et al. reported that the size of the family was significantly different between cases and controls, however there was no mention of whether this could have an influence on the results [32]. According to a systematic review on water, hygiene and sanitation, the size of the households is probably a confounding factor [38]. Spiegel et al. grouped the refugee camps with the same amount of water; therefore it remains unclear if they used appropriate eligibility criteria, as hygiene parameters can influence the living conditions of the refugees [34,38]. The study of Roberts et al. grouped the participants with the same amount of water. However, it is not clear if these groups also included participants with an improved bucket, since this can influence the diarrhoeal incidence, especially in children younger than 5 [33]. Inappropriate methods for measuring exposure and outcome variables were found in 5 studies [31,33–36] because some studies used a questionnaire to interrogate the participants, which can result in recall bias [31,33,36]. One study used the total amount of water supplied to the camp and divided it by the total number of refugees [34], while another study calculated the quantity of water consumed by cases and controls [35]. In both studies the water amount is only estimated and therefore not the most appropriate method.

None of the studies controlled for all the possible confounders (communal latrine coverage, distance from the farthest dwelling to water point, number of people at each water point…), however some studies controlled for some confounding factors [34,36]. The study by Hatch et al. used a multivariate analysis, but it is not clear for which factors the results were adjusted [32]. In two studies by Hatch et al. and Roberts et al. it was not clear if there was a complete or adequate follow-up [32,33]. Because of all these limitations there was reason to downgrade the level of evidence by one level.

The level of evidence was also downgraded because of imprecision, due either to a large variability in the results [34] or incomplete data [33,35,36]. We did not downgrade the level of evidence for inconsistency or indirectness. The limited number of studies made it difficult to evaluate publication bias. Because of limitations in design in all studies, there was no reason to upgrade the strength of the body of evidence, which based on the GRADE approach, is very low. This in turn means that every estimate of effect is very uncertain.

## Discussion

### Summary of findings

The aim of this study was to collect the available evidence for the amount of water that is needed for drinking, cooking and hygienic purposes, according to the rules of Evidence-Based Practice. After a systematic literature review and a quality assessment of the available evidence using GRADE, 6 observational studies were identified, but we found no evidence for the Sphere indicator or for any of the other standards in use. However, although no unambiguous evidence was found, receiving a greater amount of water was related to a decrease in diarrhoeal incidents [31] and to a decrease in mortality rates in children younger than 5 [34]. Another study indicated that having water containers reduced the risk of cholera [32]. Due to insufficient data, the other 2 studies did not show any significant evidence about the amount of water received and associated health effects [33,36] nor about the effect of possessing a water container [33].

### Limitations of the systematic review

There are several limitations in this systematic review. First of all, evidence is scarce concerning the chosen subject: from 3630 potentially relevant studies, 111 studies were relevant to the PICO question, of which only 6 studies met the selection criteria. Moreover, the 6 included studies show serious heterogeneity, since different populations, water supply, outcomes, and contexts were described and studied. Only 1 of these 6 studies was performed during the disaster, which indicates the lack of evidence in this phase. The 5 other studies were performed in refugee camps in the post-emergency phase. Refugee camps were included because such camps are often installed after major disasters and because it is more likely that research is done once the acute phase of a disaster has passed. There are several reasons for the low number of published studies concerning this subject in particular, and disaster aid in general: difficulties with data collection and study designs in disaster settings, lack of funding, ethical considerations, and lack of reporting and data publishing [7–9,39]. A second limitation is that the quality of the evidence available in the 6 included studies was very low. Four out of the 6 studies were cross-sectional study designs, which can never establish causality between exposures and outcomes.

### Opportunities for further research

Only one study was performed in a direct disaster setting, which clearly indicates a gap in evidence. For some interventions it is possible to investigate effectiveness in settings that differ from a disaster setting, such as interventions that reduce water contamination. However, other water and sanitation interventions should be investigated in actual disaster settings, in order to evaluate their potential real-life benefits overall [31]. Therefore, further research during and in the aftermath of disasters is necessary, not only on the amount of domestic water used but on the different components separately as well [22]. Such research is not impossible: observational and intervention studies during disasters are feasible, on condition that funding, human resources, logistical, safety and practical difficulties are considered before research is undertaken [10,40].

### From evidence to evidence-based indicator

In order to be able to define an evidence-based indicator, more research is needed on this topic [22]. Only when more evidence is available, can an evidence-based guideline be developed by placing the available evidence in context and collecting practical experience and expert opinion from those working in the field, e.g. humanitarian aid workers and physiologists.

Factors to be taken into account when developing an indicator include: the difference between having access to and actually using a certain amount of water, demographics (age, gender) and physiological determinants (metabolic rate, exercise), environmental factors (e.g. temperature, humidity), socio-cultural aspects, type and phase of the disaster (acute vs. post-emergency). Since these factors will definitely influence the amount of water needed, it would be even better to specify different minimum water requirements for different situations, types of disasters, climates. However, based on the available evidence, this is currently not possible.

For the factor concerning “phase of the disaster”, there is disagreement among experts in the field. According to Spiegel et al. an amount of 15 l/p/d would be sufficient in the acute phase of a disaster, and this amount should be increased to 20 l/p/d or even a higher amount in the post-emergency phase of the disaster [34]. However, other authors are convinced that in the acute phase of a disaster, at least 15–20 l/p/d should be supplied, and once people have settled in a camp they may survive on less [37].

In conclusion, evidence on this crucial topic during disasters is lacking and more primary research is definitely necessary if the humanitarian sector wants to further professionalise its handling of disasters and reduce the suffering of those affected as much as possible with the current best knowledge about the effectiveness of humanitarian aid interventions [9,26].

## Supporting Information

S1 Appendix PRISMA checklist. (PDF)

S2 Appendix Search strategies. (PDF)

S3 Appendix List of studies excluded, including the reason for exclusion. (PDF)
