# Supplementary material for: A Systematic Review of the Amount of Water per Person per Day Needed to Prevent Morbidity and Mortality in (Post-)Disaster Settings
Source: PLoS One. 2015 May 11;10(5):e0126395. doi: 10.1371/journal.pone.0126395 (PMC4427459; doi:10.1371/journal.pone.0126395)
Supplement: S2 Appendix — (PDF) [file pone.0126395.s002.pdf]

Supporting information

Appendix 2 Search strategies

The following search formula was used for searching MEDLINE:

1. "Disasters"[Mesh] OR Disaster*[TIAB]
2. "Droughts"[Mesh] OR drought*[TIAB] OR "Cyclonic Storms"[Mesh] OR hurricane*[TIAB] OR "Floods"[Mesh] OR flood*[TIAB] OR "Fires"[Mesh] OR forest fire*[TIAB]
3. "Geological Processes"[Mesh] OR tidal wave*[TIAB] OR volcanic eruption*[TIAB] OR tsunami*[TIAB] OR landslide*[TIAB] OR avalanche*[TIAB] OR earthquake*[TIAB] OR Storm*[TIAB] OR wildfire*[TIAB] OR Snowstorm*[TIAB] OR heat wave*[TIAB] OR cold wave*[TIAB] OR land fire*[TIAB] OR Sandstorm*[TIAB] OR cyclone*[TIAB] OR typhoon*[TIAB] OR "Extreme Heat"[Mesh] OR "Extreme Cold"[Mesh] OR extreme heat*[TIAB] OR extreme cold*[TIAB] OR extreme temperature*[TIAB]
4. "Refugees"[Mesh] OR Camp[TIAB] OR camps[TIAB] OR refugee*[TIAB] OR internally displaced[TIAB]
5. 1-4 OR
6. "Water"[Mesh] OR "Drinking Water"[Mesh] OR "Drinking"[Mesh]
7. Water amount*[TIAB] OR water quantit*[TIAB] OR (availab* AND water[TIAB]) OR (Drink* AND water[TIAB])
8. "Water Supply"[Mesh:noexp] OR water suppl*[TIAB]
9. "Water Deprivation"[Mesh] OR "Sanitation"[Mesh] OR water shortage*[TIAB] OR water deprivation*[TIAB] OR water scarcity[TIAB]
10. 6-9 OR
11. "Diarrhea"[Mesh] OR diarrhea*[TIAB] OR diarrhoea*[TIAB] OR "Cholera"[Mesh] OR cholera[TIAB] OR “Dysentery”[Mesh] OR dysentery[TIAB]
12. "Mortality"[Mesh] OR "Morbidity"[Mesh] OR infection*[TIAB] OR infectious*[TIAB] OR dehydration[TIAB]
13. "Communicable Diseases"[Mesh] OR microorganism*[TIAB] OR parasite*[TIAB] OR virus*[TIAB] OR pathogen*[TIAB] OR bacteria[TIAB] OR disease[TIAB] OR diseases[TIAB]

14. "Quality of Life"[Mesh] OR "Health Status"[Mesh] OR "Health Status Indicators"[Mesh] OR "Health"[Mesh] OR "Malaria"[Mesh] OR "Respiratory Tract Diseases"[Mesh] OR "Delivery of Health Care"[Mesh]
15. 11-14 OR
16. 5 AND 10 AND 15

The following search formula was used in EMBASE:

1. 'disaster'/exp OR disaster*:ab,ti
2. 'avalanche'/exp OR avalanche*:ab,ti OR 'earthquake'/exp OR earthquake*:ab,ti OR 'tsunami'/exp OR tsunami*:ab,ti OR 'drought'/exp OR drought*:ab,ti OR 'volcano'/exp OR (volcanic NEXT/1 eruption*):ab,ti OR 'landslide'/exp OR landslide*:ab,ti
3. flood*:ab,ti OR (forest NEXT/1 fire*):ab,ti OR (tidal NEXT/1 wave*):ab,ti OR hurricane*:ab,ti OR (land NEXT/1 fire*):ab,ti OR (cold NEXT/1 wave*):ab,ti OR (heat NEXT/1 wave*):ab,ti OR sandstorm*:ab,ti OR snowstorm*:ab,ti OR wildfire*:ab,ti OR storm*:ab,ti OR cyclone*:ab,ti OR typhoon*:ab,ti OR (extreme NEXT/1 cold*):ab,ti OR (extreme NEXT/1 heat*):ab,ti OR (extreme NEXT/1 temperature*):ab,ti
4. 'refugee'/exp OR camps:ab,ti OR camp:ab,ti OR refugee*:ab,ti OR (internally NEXT/1 displaced):ab,ti
5. 1-4 OR
6. 'water'/de OR 'water standard'/exp OR 'drinking water'/exp OR 'drinking'/exp
7. (water:ab,ti AND amount*:ab,ti) OR (water:ab,ti AND quantit*:ab,ti) OR (drink*:ab,ti AND water:ab,ti) OR (availab*:ab,ti AND water:ab,ti)
8. 'water supply'/exp OR 'tap water'/exp OR (water:ab,ti AND suppl*:ab,ti)
9. 'water deprivation'/exp OR 'sanitation':ab,ti OR (water:ab,ti AND shortage*:ab,ti) OR (water NEXT/1 deprivation*):ab,ti OR (water NEXT/1 scarcity):ab,ti
10. 6-9 OR
11. 'diarrhea'/exp OR diarrohea*:ab,ti OR diarrhea*:ab,ti OR diarrhoea*:ab,ti OR 'cholera'/exp OR 'dysentery'/exp OR cholera:ab,ti OR dysentery:ab,ti
12. 'mortality'/exp OR 'morbidity'/exp OR infectious*:ab,ti OR infection*:ab,ti OR dehydration:ab,ti
13. 'communicable disease'/exp OR pathogen*:ab,ti OR virus*:ab,ti OR parasite*:ab,ti OR microorganism*:ab,ti OR bacteria:ab,ti OR diseases:ab,ti OR disease:ab,ti

14. 'quality of life'/exp OR 'health status'/exp OR 'health survey'/exp OR 'health'/exp OR 'malaria'/exp OR 'respiratory tract disease'/exp OR 'health care delivery'/exp
15. 11-14 OR
16. 5 AND 10 AND 15

In The Cochrane Library the following search formula was used:

1. [mh Disasters] OR disaster*:ti,ab,kw
2. [mh Floods] OR [mh Fires] OR [mh "Cyclonic Storms"] OR forest fire*:ti,ab,kw OR tidal wave*:ti,ab,kw OR hurricane*:ti,ab,kw OR drought*:ti,ab,kw
3. [mh "Geological Processes"] OR avalanche*:ti,ab,kw OR landslide*:ti,ab,kw OR tsunami*:ti,ab,kw OR volcanic eruption*:ti,ab,kw OR earthquake*:ti,ab,kw OR cyclone*:ti,ab,kw OR [mh "extreme heat"] OR extreme heat*:ti,ab,kw OR [mh "extreme cold"] OR extreme cold*:ti,ab,kw OR extreme temperature*:ti,ab,kw
4. [mh Refugees] OR camp:ti,ab,kw OR camps:ti,ab,kw OR refugee*:ti,ab,kw OR internally displaced:ti,ab,kw
5. 1 - 4 OR
6. [mh Water] OR water amount*:ti,ab,kw OR water shortage*:ti,ab,kw OR water scarcity:ti,ab,kw
7. [mh Drinking] OR [mh "Drinking Water"] OR drinking water:ti,ab,kw OR drinking:ti,ab,kw
8. [mh "Water Supply"] OR water suppl*:ti,ab,kw
9. [mh Sanitation] OR [mh "Water Deprivation"] OR water deprivation:ti,ab,kw
10. 6 - 9 OR

5 AND 10
