# Supplementary material for: A Systematic Review of the Amount of Water per Person per Day Needed to Prevent Morbidity and Mortality in (Post-)Disaster Settings
Source: PLoS One. 2015 May 11;10(5):e0126395. doi: 10.1371/journal.pone.0126395 (PMC4427459; doi:10.1371/journal.pone.0126395)
Supplement: S3 Appendix — (PDF) [file pone.0126395.s003.pdf]

Supporting information

Appendix 3 Table of excluded studies and reason for exclusion

Reference	Reason for exclusion	
Abbott D et al., 2000	Design	Overview of interventions in California
Abouteir A et al., 2011	Intervention	Amount of water was not mentioned
Abu Mourad TA., 2004	Intervention	Amount of water was not mentioned
Aitken et al., 2010	Intervention	Amount of water was not mentioned
Allwood P, et al, 2014	Intervention	Amount of water was not mentioned
Amato RL et al., 2001	Intervention	Amount of water was not mentioned
Anand JK, 1995	Design	Comment/letter
Atuyambe LM et al., 2011	Intervention	Total amount of water was not mentioned
Barnes J, 1971	Design	Correspondence
Bechen R. et al. 1996	Design	Opinion pieces
Berro et al., 2004	Intervention	Amount of water was not mentioned
Bhave SY, et al, 2005	Intervention	Amount of water was not mentioned
Bhunia R, et al, 2011	Intervention	Amount of water was not mentioned
Bile K.M et al., 2010	Intervention	Amount of water was not mentioned
Brana AM, et al, 2013	Design	Conference abstract
Brodie M et al., 2006	Intervention	Amount of water was not mentioned
Brown C et al., 2011	Intervention	Amount of water was not mentioned
Brown CM et al, 2014	Design	Editorial
Brown V, et al, 2002	Design	Narrative review
Burki T, 2013	Design	Newsdesk WHO
Bwire G, et al, 2013	Intervention	Water supply not mentioned
Campuzano CP, et al, 2014	Language	Spanish
Carmichael C, et al, 2013	Intervention	Amount of water was not mentioned
Centers for Disease Control and Prevention (CDC) 2011	Intervention	Amount of water was not mentioned
Centers for Disease Control and prevention (CDC), 2003	Design	Weekly report
Cerda R, et al, 2013	Design	Comment
Chin SH, et al, 2013	Design	Conference abstract
Counts CS et al., 2001	Intervention	Water supply was not mentioned
Craig-Gray R, 2012	Design	Case report
Cronin AA et al., 2009	Design	No comparison
Cyranoski D, 2011	Design	News report
De Ville de Goyet C et al., 2001	Language	Spanish
Deebaj R et al., 2011	Intervention	Amount of water was not mentioned
Doocy S et al., 2006	Intervention	Disinfectants compared with no disinfectants
Drazen JM et al, 2005	Design	Editorial
Drysdale S et al., 2000	Intervention	Not objective on water amount
Dunkle SE, et al, 2011	Design	Conference abstract
Englande AJ Jr., 2008	Intervention	Amount of water was not mentioned
Ferrier et al., 2007	Design	Opinion piece
Frist WH, et al, 2005	Design	Perspective
Gambhir RS, et al, 2013	Other	Full text not available
Gelting R, et al, 2013	Design	Narrative review
Gerald BL, 2005	Intervention	Amount of water was not mentioned

Grandesso F, et al, 2014	Intervention	Amount of water was not mentioned
Gulland A. et al., 2012	Design	Opinion piece
Holt E, et al, 2014	Design	News
Howie et al., 1979	Design	Interview
Hurlimann A.C et al., 2009	Design	Review on willingness to pay for water
Jafari N. et al., 2011	Intervention	Amount of water was not mentioned
Jafri T.K et al., 2012	Intervention	Amount of water was not mentioned
Javaid C, et al, 2012	Design	general review
Karmakar S et al., 2008	Intervention	Amount of water was not mentioned
Khan MU, et al, 1982	Intervention	Amount of water was not mentioned
Kirsch TD, et al, 2010	Intervention	Amount of water was not mentioned
Krcmery V et al., 2010	Intervention	Amount of water was not mentioned
Kumar T, et al, 2013	Design	Conference abstract
Kunii O et al., 2002	Intervention	Amount of water was not mentioned
Kwanbunjan K et al., 2006	Intervention	Amount of water was not mentioned
Lantagne D, et al, 2013	Intervention	Amount of water was not mentioned
Lantagne D, et al, 2014	Design	Study on the source of cholera
Li S et al., 1998	Language	Chinese
Ligon BL, 2006	Design	Review, do not mentioned the amount of water
Lim JH, 2005	Intervention	Amount of water was not mentioned
Linscott A.J. et al., 2007	Intervention	Amount of water was not mentioned
Loewenberg S, et al, 2014	Design	News report
Lumley J, et al, 2006	Design	Editorial
Malilay J. et al., 2000	Design	Review on multidisciplinary approach
Moll DM et al., 2007	Intervention	Amount of water was not mentioned
Mondal et al., 2001	Intervention	Amount of water was not mentioned
Moors E, et al, 2013	Population	Study on climat change
Noji et al., 2005	Design	Opinion piece
Onishchenko G.G et al., 2001	Language	Russian
Oswald WE et al., 2008	Design	No comparison
Parmar P, et al, 2013	Intervention	Study on emergency care and disaster response
Pawar AT et al., 2005	Intervention	Amount of water was not mentioned
Pfrimmer DM, 2010	Design	Clinical update
Pinera JF et al., 2009	Outcome	Health effects were not mentioned
Rapaic M, 1972	Language	Croatian
Rashid SF, 2009	Intervention	Amount of water was not mentioned
Rebaudet S, et al, 2013	Intervention	Amount of water was not mentioned
Schuller M, et al, 2014	Intervention	Amount of water was not mentioned
Scobie H, et al, 2013	Design	Conference abstract
Shabir O, 2013	Intervention	Amount of water was not mentioned
Smilkstein G, 1981	Design	Narrative review
Sorenson S.B. et al., 2011	Intervention	Amount of water was not mentioned
Sumner SA, et al, 2013	Intervention	Amount of water was not mentioned
Tamason C, et al, 2013	Design	Conference abstract
Tappero JW et al., 2011	Design	Opinion piece
Tokuda K, et al, 2014	Intervention	Amount of water was not mentioned
Toole M.J. et al., 1995	Intervention	Amount of water was not mentioned
Toole M.J. et al., 1997	Design	Review on public health effects
Ueda S et al., 2012	Intervention	Amount of water was not mentioned

Van Poppel et al., 1997	Design	Narrative review
Vandevelde T et al., 2002	Design	Opinion piece
Venturi L et al., 2011	Design	Case study
Wampler P, et al, 2011	Design	Correspondence
Wang G.-S. et al., 2001	Language	Chinese
Waring SC et al., 2005	Intervention	Amount of water was not mentioned
Watson J.T et al., 2007	Design	Review on risk factors for disease outbreaks
Wilderer PA, 2004	Design	Review on interventions
Wisitwong A et al., 2010	Intervention	Amount of water was not mentioned
Woersching JC et al., 2003	Intervention	Amount of water was not mentioned
Wu J et al., 2011	Intervention	Amount of water was not mentioned
Zabaneh JE et al., 2008	Intervention	Amount of water was not mentioned
Zeng et al., 2008	Design	Opinion piece; narrative review
Zhou Y, et al, 2013	Language	Chinese
